# Breast cancer detection accuracy of AI in an entire screening population: a retrospective, multicentre study

**DOI:** 10.1186/s40644-023-00643-x

**Published:** 2023-12-20

**Authors:** Mohammad Talal Elhakim, Sarah Wordenskjold Stougaard, Ole Graumann, Mads Nielsen, Kristina Lång, Oke Gerke, Lisbet Brønsro Larsen, Benjamin Schnack Brandt Rasmussen

**Affiliations:** 1 Department of Radiology, Odense University Hospital, Kløvervaenget 47, Entrance 27, Ground floor, 5000 Odense C, Denmark; 2 Department of Clinical Research, University of Southern Denmark, Kløvervaenget 10, Entrance 112, 2nd floor, 5000 Odense C, Denmark; 3 Department of Radiology, Aarhus University Hospital, Palle Juul-Jensens Blvd. 99, 8200 Aarhus N, Denmark; 4 Department of Clinical Research, Aarhus University, Palle Juul-Jensens Blvd. 99, 8200 Aarhus N, Denmark; 5 Department of Computer Science, University of Copenhagen, Universitetsparken 1, 2100 København Ø, Denmark; 6 Department of Translational Medicine, Lund University, Inga Maria Nilssons gata 47, SE-20502 Malmö, Sweden; 7 Unilabs Mammography Unit, Skåne University Hospital, Jan Waldenströms gata 22, SE-20502 Malmö, Sweden; 8 Department of Nuclear Medicine, Odense University Hospital, Kløvervaenget 47, Entrance 44, 5000 Odense C, Denmark; 9 CAI-X – Centre for Clinical Artificial Intelligence, Odense University Hospital, Kløvervaenget 8C, Entrance 102, 5000 Odense C, Denmark

**Keywords:** Artificial intelligence, Deep learning, Breast cancer, Mammography screening, Double reading

## Abstract

**Background:**

Artificial intelligence (AI) systems are proposed as a replacement of the first reader in double reading within mammography screening. We aimed to assess cancer detection accuracy of an AI system in a Danish screening population.

**Methods:**

We retrieved a consecutive screening cohort from the Region of Southern Denmark including all participating women between Aug 4, 2014, and August 15, 2018. Screening mammograms were processed by a commercial AI system and detection accuracy was evaluated in two scenarios, Standalone AI and AI-integrated screening replacing first reader, with first reader and double reading with arbitration (combined reading) as comparators, respectively. Two AI-score cut-off points were applied by matching at mean first reader sensitivity (AI_sens_) and specificity (AI_spec_). Reference standard was histopathology-proven breast cancer or cancer-free follow-up within 24 months. Coprimary endpoints were sensitivity and specificity, and secondary endpoints were positive predictive value (PPV), negative predictive value (NPV), recall rate, and arbitration rate. Accuracy estimates were calculated using McNemar’s test or exact binomial test.

**Results:**

Out of 272,008 screening mammograms from 158,732 women, 257,671 (94.7%) with adequate image data were included in the final analyses. Sensitivity and specificity were 63.7% (95% CI 61.6%-65.8%) and 97.8% (97.7-97.8%) for first reader, and 73.9% (72.0-75.8%) and 97.9% (97.9-98.0%) for combined reading, respectively. Standalone AI_sens_ showed a lower specificity (-1.3%) and PPV (-6.1%), and a higher recall rate (+ 1.3%) compared to first reader (*p* < 0.0001 for all), while Standalone AI_spec_ had a lower sensitivity (-5.1%; p < 0.0001), PPV (-1.3%; p = 0.01) and NPV (-0.04%; p = 0.0002). Compared to combined reading, Integrated AI_sens_ achieved higher sensitivity (+ 2.3%; p = 0.0004), but lower specificity (-0.6%) and PPV (-3.9%) as well as higher recall rate (+ 0.6%) and arbitration rate (+ 2.2%; p < 0.0001 for all). Integrated AI_spec_ showed no significant difference in any outcome measures apart from a slightly higher arbitration rate (*p* < 0.0001). Subgroup analyses showed higher detection of interval cancers by Standalone AI and Integrated AI at both thresholds (*p* < 0.0001 for all) with a varying composition of detected cancers across multiple subgroups of tumour characteristics.

**Conclusions:**

Replacing first reader in double reading with an AI could be feasible but choosing an appropriate AI threshold is crucial to maintaining cancer detection accuracy and workload.

**Supplementary Information:**

The online version contains supplementary material available at 10.1186/s40644-023-00643-x.

## Background

Early detection with mammography screening along with best practice treatment are recognized as crucial elements in reducing breast cancer-specific mortality and morbidity [1], and most European and high-income countries have implemented organised mammography screening programmes [2, 3]. The rollout of the Danish screening programme for women aged 50–69 years was completed in 2010, and the programme has shown high compliance with international standards [4, 5], based on quality assurance indicators in conformity with European guidelines [6]. However, widespread capacity issues and shortage of breast radiologists propose a threat to the continued feasibility and efficiency of the screening programme. Addressing these challenges, The Danish Health Authority has recommended replacing first reading breast radiologists in the double reading setting with an artificial intelligence (AI) system, if shown efficient [7].

Deep learning-based AI decision support systems have in recent years gained popular interest as a potential solution to resource scarcity within mammography screening as well as improving cancer detection. Strong claims have been made that an AI system could replace trained radiologists [8, 9]. Multiple validation studies have reported a standalone AI cancer detection accuracy at a level comparable to or even exceeding current standard for breast cancer screening [10–12]. While the results might seem promising, these are yet to be replicated in large real-life screening populations. Moreover, the quantity and quality of the existing evidence has been deemed insufficient [13], and recent guidelines by the European Commission Initiative on Breast Cancer have recommended against single reading supported with AI [14].

In this external validation study, we aimed to investigate the accuracy of a commercially available AI system for cancer detection in a Danish mammography screening population with at least two years of follow-up. The AI system was evaluated both in a simulated Standalone AI scenario and a simulated AI-integrated screening scenario replacing first reader, compared with the first reader and double reading with arbitration.

## Methods

### Study design and population

This study was designed as a retrospective, multicentre study on the accuracy of an AI system for breast cancer detection in mammography screening. The study is reported in accordance with Standards for Reporting of Diagnostic Accuracy Studies (STARD) statement of 2015 (Supplementary eMethod 1) [15]. Ethical approval was obtained from the Danish National Committee on Health Research Ethics (identifier D1576029) which waived the need for individual informed consent.

The study population was a consecutive cohort from all breast cancer screening centres in the Region of Southern Denmark (RSD) in the cities Aabenraa, Esbjerg, Odense, and Vejle. The study sites cover all the RSD, one of five Danish regions, with approximately 1.2 million inhabitants, comprising 20% of the entire population of Denmark and constituting an entire screening population.

All women who participated in screening between Aug 4, 2014, and Aug 15, 2018, in RSD were eligible for inclusion. The majority were women between 50 and 69 years participating in the standardised two-year interval screening programme. A small group with previous breast cancer or genetic predisposition to breast cancer were biennially screened from the age of 70–79 years or from 70 years of age until death, respectively.

Exclusion criteria were insufficient follow-up until cancer diagnosis, next consecutive screening, or at least two years after the last performed screening in the inclusion period, insufficient image quality or lacking images, and unsupported data type by the AI system.

### Data sources and extraction

A complete list of the study population including reader decisions and site of screening was locally extracted from the local Radiological Information System using the study participants’ unique Danish Civil Personal Register numbers. Image data was extracted in raw DICOM format from the joint regional radiology Vendor Neutral Archive. All screening examinations had been acquired with a single mammography vendor, Siemens Mammomat Inspiration (Siemens Healthcare A/S, Erlangen, Germany). The standard screening examination was two views per breast, but could be less, e.g. in case of prior mastectomy, or more if additional images were taken, e.g. due to poor image quality.

Information on cancer diagnosis and histological subtype, with tumour characteristics for invasive cancers including tumour size, malignancy grade, TNM stage, lymph node involvement, estrogen receptor (ER) status, and HER2 status, was acquired through matching with the Danish Clinical Quality Program – National Clinical Registries (RKKP), specifically the Danish Breast Cancer Cooperative Group database and the Danish Quality Database on Mammography Screening [4, 16]. Inconsistencies in the data were, if possible, resolved by manually searching the electronic health records.

### Screen reading

The screen reading consisted of independent, blinded double reading by 22 board-certified breast radiologists with experience in screen reading ranging from newly trained to over 20 years of experience. There was no fixed designation of the readers, however, the second reader is usually a senior breast radiologist. The reading assessments were ultimately classified into a binary outcome: either normal (continued screening) or abnormal (recall). Cases of disagreement were sent to a decisive third reading, i.e. arbitration, by the most experienced screening radiologist who had access to the first two readers’ decisions, although the arbitrator could also have been second reader of the same examination. Diagnostic work-up of recalled women was performed at dedicated breast imaging units at the study sites.

### AI system

As index test for this study, we used the commercially available CE marked and FDA cleared AI system Transpara version 1.7.0 (ScreenPoint Medical BV, Nijmegen, Netherlands), a software-only device based on deep convolutional neural networks intended for use as concurrent reading aid for breast cancer detection on mammography. The model was trained and tested using large databases acquired through multivendor devices from institutions across the world [10, 17]. The data used in this study has never been used for training, validation or testing of any AI models.

Transpara was installed on an on-premises dedicated server system to which only the local investigators had access. All screening mammograms meeting Transpara’s DICOM conformance statement were sent for processing. Transpara assigned a per-view regional prediction score from 1 to 98 denoting the likelihood of cancer, with 98 indicating the highest likelihood of the finding being malignant. The maximum of the view-level raw scores gave a total examination score, Transpara exam score, on a scale from 0 to 10 with five decimal points.

### Evaluation scenarios

**Fig. 1:**
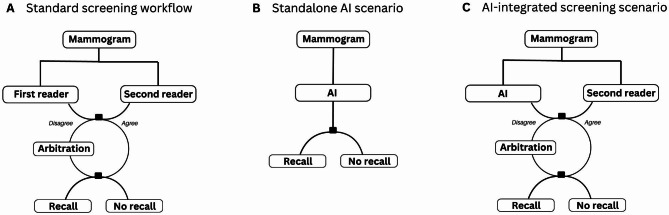
Comparison between the standard screening workflow and the study scenarios **(A)** The standard screening workflow in which the combined reading outcome of each mammogram is the result of independent, blinded double reading with arbitration for discordant readings. **(B)** The Standalone AI scenario in which the AI system replaces all readers, and the AI detection accuracy is compared to that of the first reader in the study sample. **(C)** The AI-integrated screening scenario in which AI replaces the first reader in the standard screening workflow, and the detection accuracy of the simulated screening setup is compared to that of the combined reading outcome from the study sample. In both study scenarios **(A)** and **(B)**, a binary AI score was defined by applying two different thresholds for the AI decision outcome. The cut-off points were chosen by matching at the mean sensitivity and specificity of the first reader outcome, AI_sens_ and AI_spec_, respectively. If the AI and second reader decisions were discordant in the AI-integrated screening scenario and an arbitration decision was lacking in the original dataset, the arbitration decision outcome was simulated to match the same accuracy level of the original arbitrator from the study sample

The detection accuracy of the AI system was assessed in two scenarios: (1) “Standalone AI” where AI accuracy was evaluated against that of the first reader, and (2) “AI-integrated screening”, a simulated screening setup, in which the AI replaced the first reader, compared against the combined reading outcome, i.e. the observed screen reading decision of double reading with arbitration in the standard screening workflow without AI (Fig. 1). In the AI-integrated screening scenario, the original decisions of the second reader and arbitrator were applied. In cases of disagreement between the AI and second reader, where an arbitration was not originally performed at screening, a simulated arbitrator was defined with arbitration decisions at an accuracy level which approximated the original arbitrator’s sensitivity and specificity from the study sample. These simulated arbitration decisions were applied as the arbitration outcome in cases lacking an original arbitration decision.

As the AI system is not intended for independent reading and does not have an internally prespecified threshold to classify images, the Transpara exam score was in both scenarios dichotomized into an AI score that would enable comparability with the radiologists. In this study, two different thresholds were explored as test abnormality cut-off points, AI_sens_ and AI_spec_, which were set to match the mean sensitivity and specificity, respectively, of the first reader outcome from the study sample. Outcomes above the threshold were considered as recalls. There is a lack of consensus in the literature on how to determine an appropriate test threshold [13], but by matching the cut-off point at the first reader’s sensitivity or specificity, this would hypothetically ensure that the proposed AI-integrated screening would not entail an increase in false positive recalls or missed cancers, respectively, which could be clinically justifiable in screening practice.

### Performance metrics and reference standard

In both scenarios, the measures of detection accuracy were sensitivity and specificity as coprimary endpoints, and positive predictive value (PPV), negative predictive value (NPV), recall rate, and arbitration rate as secondary endpoints. The reference standard for positive cancer outcome was determined through histopathological verification of breast malignancy including non-invasive cancer, i.e. ductal carcinoma in situ, at screening (screen-detected cancer) or up until the next consecutive screening within 24 months (interval cancer). The reference standard for negative cancer outcome was defined as cancer-free follow-up until the next consecutive screening or within 24 months. The choice of a two-years’ follow-up period for the reference standard concords with that commonly used in cancer registries and quality assessment of biennial screening programmes. However, breast cancer can be present long before it is diagnosed [18], and diagnostic work-up of AI-recalled cases is not performed to confirm the presence of such potential cancers. To take this potential bias into account and to investigate for early detection patterns, an exploratory analysis of detection accuracy was performed with inclusion of next-round screen-detected cancers (diagnosed in the subsequent screening) and long-term cancers (diagnosed > 2–7 years after screening).

### Statistical analysis

Binomial proportions for the accuracy of AI and radiologists were calculated and supplemented by 95% Clopper-Pearson (‘exact’) confidence intervals (CI). AI accuracy was compared to that of radiologists using McNemar’s test or exact binomial test when discordant cells were too small. Accuracy analysis of all outcomes across radiologist position is presented in the supplementary material (eTable 1). To examine consistency of the AI accuracy among subgroup variables, detection rates were calculated by cancer subgroups. Furthermore, detection agreements and discrepancies between the radiologists and AI were investigated across cancer subgroups (Supplementary eTables 2–3). A *p* value of less than 0.05 was considered statistically significant. Stata/SE 17 (College Station, Texas 77,845 USA) was used for data management and analyses.

## Results

### Study sample and characteristics

**Fig. 2:**
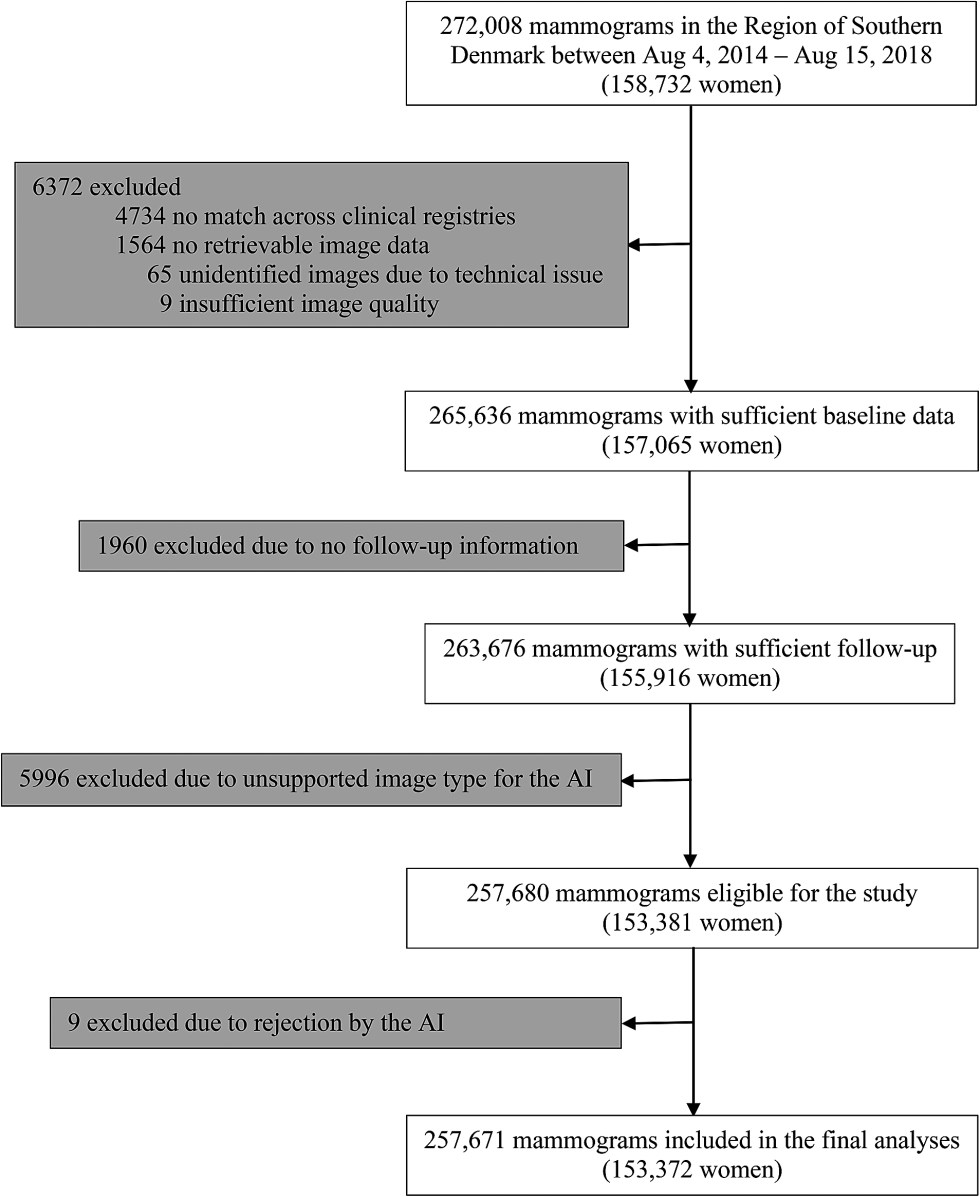
Study flow diagram The study cohort covers an entire mammography screening population across two successive biennial screening rounds, for which reason most women contribute with more than one screening mammogram to the cohort. AI = artificial intelligence

We retrieved a total of 272,008 unique screening mammograms from 158,732 women in the study population, among which 14,337 (5.3%) were excluded from the analyses (Fig. 2).

The characteristics of the 257,671 mammograms included in the analyses are summarised in Table 1. The cancer prevalence in the sample was 2014 (0.8%) of which 1517 (74.3%) were screen-detected, yielding a detection rate of 5.9 per 1000 screening mammograms and a recall rate of 2.7%.

**Table 1:** Clinical characteristics of the study sample

	Study sample (n = 257,671)
Screening site	
Aabenraa Esbjerg Odense Vejle	49,641 (19.3) 49,860 (19.4) 104,984 (40.7) 53,186 (20.6)
Age at screening, years	
< 50^*^ 50–59 60–69 70–79 ≥ 80	59.3 (6.0) 25 (< 0.1) 133,223 (51.7) 120,315 (46.7) 4024 (1.6) 84 (< 0.1)
Breast cancer prevalence Screen-detected cancer Interval cancer	2041 (0.8) 1479 (72.5) 562 (27.5)
Breast cancer type	
Invasive cancer DCIS	1830 (89.7) 211 (10.3)
Screening outcome^†^	
Normal Abnormal	250,810 (97.3) 6861 (2.7)
Arbitrations^‡^	7434 (2.9)
Agreement between readers^‡^	
First and second reader First reader and arbitrator Second reader and arbitrator	250,663 (97.3) 3299 (44.4) 4537 (61.0)

Data are n (%) or mean (SD). DCIS = ductal carcinoma in situ

*These women were all 49 years old and were invited to regular biennial screening a few months too early

†Combined reading outcome of the double reading with arbitration

‡There is a small overlap of n = 426 (0.2%) studies in the arbitrations and agreements between first and second readers due to disagreements on subset outcomes with additional initiatives, such as stereotactic breast biopsy, which were eventually classified into the available binary screening outcome

The accuracy of the first reader in terms of sensitivity and specificity was 63.7% (95% CI 61.6%-65.8%) and 97.8% (97.7-97.8%), respectively (Table 2), which was used to choose the thresholds for the AI score. Hence, AI_sens_ and AI_spec_ used a Transpara exam score of 9.56858 and 9.71059, respectively. The distribution of the Transpara exam scores across the study sample has been visualised in the supplementary material (eFigure 1). The accuracy of the combined reading in terms of sensitivity and specificity was 73.9% (95% CI 72.0%-75.8%) and 97.9% (97.9-98.0%), respectively. The accuracy analysis across coprimary and secondary outcomes in both study scenarios is described in Table 2. Moreover, a comparison between the screening outcome and the reference standard (true and false positives and negatives) in both study scenarios, along with a descriptive workload analysis, is presented in the supplementary material (eTable 4).

**Table 2:** Detection accuracy analysis in both study scenarios

	Sensitivity (95% CI); *p* value*	Specificity (95% CI); *p* value*	PPV (95% CI); *p* value^†^	NPV (95% CI); *p* value^†^	Recall rate (95% CI); *p* value^†^	Arbitration rate (95% CI); *p* value*
Standalone AI scenario						
First reader	63.7 (61.6–65.8); ref.	97.8 (97.7–97.8); ref.	18.7 (17.8–19.6); ref.	99.7 (99.7–99.7); ref.	2.7 (2.6–2.8); ref.	NA
Standalone AI_sens_	63.7 (61.6–65.8); >0.99	96.5 (96.4–96.5); <0.0001	12.6 (11.9–13.2); <0.0001	99.7 (99.7–99.7); 0.71	4.0 (3.9–4.1); <0.0001	NA
Standalone AI_spec_	58.6 (56.5–60.8); <0.0001	97.8 (97.7–97.8); 0.95	17.4 (16.5–18.3); 0.01	99.7 (99.6–99.7); 0.0002	2.7 (2.6–2.7); 0.24	NA
AI-integrated screening scenario						
Combined reading	73.9 (72.0-75.8); ref.	97.9 (97.9–98.0); ref.	22.0 (21.0–23.0); ref.	99.8 (99.8–99.8); ref.	2.7 (2.6–2.7); ref.	2.9 (2.8-3.0); ref.
Integrated AI_sens_	76.2 (74.3–78.0); 0.0004	97.3 (97.2–97.3); <0.0001	18.1 (17.3–19.0); <0.0001	99.8 (99.8–99.8); 0.07	3.3 (3.3–3.4); <0.0001	5.1 (5.1–5.2); <0.0001
Integrated AI_spec_	74.6 (72.6–76.4); 0.32	97.9 (97.8–97.9); 0.54	22.0 (21.0–23.0); 0.99	99.8 (99.8–99.8); 0.60	2.7 (2.6–2.7); 0.49	4.0 (3.9–4.1); <0.0001

Data are % (95% CI); *p* value. PPV = positive predictive value. NPV = negative predictive value. AI_sens_=artificial intelligence score cut-off point matched at mean first reader sensitivity. AI_spec_=artificial intelligence score cut-off point matched at mean first reader specificity. *p values were calculated using McNemar’s test. †*p* values were calculated using exact binomial test

### Standalone AI accuracy

Standalone AI_sens_ achieved a lower specificity (-1.3%) and PPV (-6.1%) and a higher recall rate (+ 1.3%) compared to first reader (*p* < 0.0001 for all). For the latter, this corresponded to 3369 (+ 48.3%) more recalls (Supplementary eTable 4). Standalone AI_spec_ obtained a lower sensitivity (-5.1%; *p* < 0.0001) and PPV (-1.3%; *p* = 0.01) than first reader, while the recall rate at 2.7% was not significantly different (*p* = 0.24). In comparison to first reader, the cancer distribution, as detailed in Table 3, showed a higher proportion of detected interval cancers for Standalone AI_sens_ by 100 (+ 17.8%) cancers and Standalone AI_spec_ by 70 (+ 12.5%) cancers, while the detection of screen-detected cancers was lower by 100 (-6.8%) and 174 (-11.8%) cancers, respectively (*p* < 0.0001 for all). Breakdowns by cancer subgroups showed the differences to be distributed across all subgroups for both screen-detected cancers and interval cancers without any evident pattern for any of the variables (Table 4). However, subgroup analyses revealed underlying detection discrepancies between first reader and the AI system with a notable number of the AI-detected cancers being missed by first reader, and vice versa (Supplementary eTable 2).

**Table 3:** Cancer detection rates in both study scenarios

	Standalone AI			AI-integrated screening		
	**First reader**	**AI**_**sens**_	**AI**_**spec**_	**Combined reading**	**Integrated AI**_**sens**_	**Integrated AI**_**spec**_
All cancers (n = 2041)	1301 (63.7); ref.	1301 (63.7); >0.99	1197 (58.7); <0.0001	1509 (73.9); ref.	1555 (76.2); 0.0004	1522 (74.6); 0.33
Screen-detected cancers (n = 1479)	1262 (85.3); ref.	1162 (78.6); <0.0001	1088 (73.6); <0.0001	1479 (100.0); ref.	1425 (96.4); <0.0001	1413 (95.5); <0.0001
Interval cancers (n = 562) < 12 months after screening (n = 170) ≥ 12 months after screening (n = 392)	39 (6.9); ref. 13 (7.7); ref. 26 (6.6); ref.	139 (24.7); <0.0001 43 (25.3); <0.0001 96 (24.5); <0.0001	109 (19.4); <0.0001 36 (21.2); <0.0001 73 (18.6); 0.0002	30 (5.3); ref. 14 (8.2); ref. 16 (4.1); ref.	130 (23.1); <0.0001 47 (27.7); <0.0001 83 (21.2); 0.0001	109 (19.4); <0.0001 41 (24.1); <0.0001 68 (17.4); <0.0001
Histological subtype						
Invasive ductal (n = 1393) Invasive lobular (n = 222) Other invasive (n = 215) Ductal carcinoma in situ (n = 211)	905 (65.0); ref. 117 (52.7); ref. 103 (47.9); ref. 176 (83.4); ref.	907 (65.1); 0.96 128 (57.7); 0.19 87 (40.5); 0.052 179 (84.8); 0.78	833 (59.8); 0.0002 114 (51.4); 0.79 79 (36.7); 0.003 171 (81.0); 0.60	1034 (74.2); ref. 143 (64.4); ref. 121 (56.3); ref. 211 (100.0); ref.	1072 (77.0); 0.001 154 (69.4); 0.03 122 (56.7); >0.99 207 (98.1); 0.13	1053 (75.6); 0.08 145 (65.3); 0.82 122 (56.7); >0.99 202 (95.7); 0.004
Tumour size*						
0–10 mm (n = 577) 11–20 mm (n = 790) 21–50 mm (n = 380) 51 + mm (n = 49) Unknown (n = 34)	399 (69.2); ref. 521 (66.0); ref. 174 (45.8); ref. 17 (34.7); ref. 14 (41.2); ref.	379 (65.7); 0.15 513 (64.9); 0.59 194 (51.1); 0.05 26 (53.1); 0.02 10 (29.4); 0.34	342 (59.3); <0.0001 475 (60.1); 0.001 177 (46.6); 0.84 23 (49.9); 0.11 9 (26.5); 0.23	496 (86.0); ref. 581 (73.5); ref. 189 (49.7); ref. 18 (36.7); ref. 14 (41.2); ref.	484 (83.9); 0.10 598 (75.7); 0.04 226 (59.5); <0.0001 24 (49.0); 0.07 16 (47.1); 0.63	482 (83.5); 0.04 583 (73.8); 0.89 216 (56.8); <0.0001 22 (44.9); 0.22 17 (50.0); 0.30^†^
Malignancy grade*						
Grade 1 (n = 507) Grade 2 (n = 815) Grade 3 (n = 358) Unknown (n = 150)	331 (65.3); ref. 520 (63.8); ref. 193 (53.9); ref. 81 (54.0); ref.	359 (70.8); 0.02 526 (64.5); 0.73 174 (48.6); 0.05 63 (42.0); 0.01	324 (63.9); 0.60 487 (59.8); 0.03 157 (43.9); 0.0002 58 (38.7); 0.001	410 (80.9); ref. 587 (72.0); ref. 202 (56.4); ref. 99 (66.0); ref.	418 (82.5); 0.23 617 (75.7); 0.001 217 (60.6); 0.01 96 (64.0); 0.58	410 (80.9); 1.00 605 (74.2); 0.04 208 (58.1); 0.33 97 (64.7); 0.73
TNM stage*						
Local (I + II) (n = 1761) Locally advanced (III) (n = 44) Distant metastasis (IV) (n = 20) Unknown (n = 5)	1105 (62.8); ref. 15 (34.1); ref. 4 (20.0); ref. 1 (20.0); ref.	1100 (62.5); 0.85 14 (31.8); 1.00 7 (35.0); 0.10^†^ 1 (20.0); 1.00^†^	1006 (57.1); <0.0001 13 (29.6); 0.73 6 (30.0); 0.63 1 (20.0); 1.00^†^	1280 (72.7); ref. 13 (30.0); ref. 4 (20.0); ref. 1 (20.0); ref.	1324 (75.2); 0.0004 16 (36.4); 0.38 7 (35.0); 0.10^†^ 1 (20.0); 1.00^†^	1296 (73.6); 0.20 16 (36.4); 0.32^†^ 7 (35.0); 0.10^†^ 1 (20.0); 1.00^†^
Lymph node positivity*						
No (n = 1340) Yes (n = 490)	840 (62.7); ref. 285 (58.2); ref.	826 (61.6); 0.48 296 (60.4); 0.38	759 (56.6); <0.0001 267 (54.5); 0.13	984 (73.4); ref. 314 (64.1); ref.	1005 (75.0); 0.05 343 (70.0); 0.0001	992 (74.0); 0.47 328 (66.9); 0.06
ER positivity*						
0% (n = 207) 1–9% (n = 98) 10–100% (n = 1514) Unknown (n = 11)	96 (46.4); ref. 46 (46.9); ref. 977 (64.5); ref. 6 (54.6); ref.	75 (36.2); 0.003 38 (38.8); 0.22 1003 (66.3); 0.20 6 (54.6); 1.00	67 (32.4); <0.0001 33 (33.7); 0.04 920 (60.8); 0.005 6 (54.6); 1.00	102 (49.3); ref. 49 (50.0); ref. 1140 (75.3); ref. 7 (63.6); ref.	100 (48.3); 0.82 57 (58.2); 0.02 1183 (78.1); 0.0002 8 (72.7); 0.76^†^	101 (48.8); 1.00 51 (52.0); 0.75 1160 (76.6); 0.08 8 (72.7); 0.76^†^
HER2 status*						
Negative (n = 1581) Positive (n = 225) Unknown (n = 24)	992 (62.8); ref. 123 (54.7); ref. 10 (41.7); ref.	986 (62.4); 0.81 128 (56.9); 0.55 8 (33.3); 0.69	902 (57.1); <0.0001 116 (51.6); 0.37 8 (33.3); 0.69	1151 (72.8); ref. 135 (60.0); ref. 12 (50.0); ref.	1194 (75.5); 0.0003 142 (63.1); 0.21 12 (50.0); 1.00	1168 (73.9); 0.15 140 (62.2); 0.33 12 (50.0); 1.00

Data are n (%); *p* value. The cancer detection rate is reported as the number of detected cancers out of the number of true cancers for the subgroup in the same row. AI_sens_=artificial intelligence score cut-off point matched at mean first reader sensitivity. AI_spec_=artificial intelligence score cut-off point matched at mean first reader specificity. TNM = tumour, node, metastasis. ER = estrogen receptor. HER2 = human epidermal growth factor receptor 2. *Reported for invasive cancers only (n = 1.830). †*p* values were calculated using exact binomial test instead of McNemar’s test due to small discordant cells

**Table 4:** Detection rates across cancer subgroups for screen-detected cancers and interval cancers in the Standalone AI scenario

	First reader	Standalone AI_sens_	Standalone AI_spec_
Screen-detected cancers (n = 1268)*			
Tumour size			
0–10 mm (n = 487) 11–20 mm (n = 574) 21–50 mm (n = 179) 51 + mm (n = 14) Unknown (n = 14)	390 (80.1); ref. 510 (88.9); ref. 161 (89.9); ref. 11 (78.6); ref. 14 (100.0); ref.	356 (73.1); 0.007 465 (81.0); 0.0001 142 (79.3); 0.007 13 (92.9); 0.33^†^ 7 (50.0); <0.0001^†^	325 (66.7); <0.0001 436 (76.0); <0.0001 137 (76.5); 0.001 13 (92.9); 0.33^†^ 6 (42.9); <0.0001^†^
Malignancy grade			
Grade 1 (n = 403) Grade 2 (n = 576) Grade 3 (n = 192) Unknown (n = 97)	323 (80.2); ref. 505 (87.7); ref. 180 (93.8); ref. 78 (80.4); ref.	335 (83.1); 0.27 449 (78.0); <0.0001 142 (74.0); <0.0001 57 (58.8); 0.001	303 (75.2); 0.06 427 (74.1); <0.0001 133 (69.3); <0.0001 54 (55.7); 0.0002
TNM stage			
Local (I + II) (n = 1253) Locally advanced (III) (n = 11) Distant metastasis (IV) (n = 4)	1071 (85.5); ref. 11 (100.0); ref. 4 (100.0); ref.	971 (77.5); <0.0001 8 (72.7); <0.0001^†^ 4 (100.0); >0.99^†^	906 (72.3); <0.0001 8 (72.7); <0.0001^†^ 3 (75.0); <0.0001^†^
Lymph node positivity			
No (n = 964) Yes (n = 304)	814 (84.4); ref. 272 (89.5); ref.	743 (77.1); <0.0001 240 (79.0); 0.0004	692 (71.8); <0.0001 225 (74.0); <0.0001
ER positivity			
0% (n = 94) 1–9% (n = 47) 10–100% (n = 1120) Unknown (n = 7)	88 (93.6); ref. 44 (93.6); ref. 948 (84.6); ref. 6 (85.7); ref.	60 (63.8); <0.0001 28 (59.6); 0.001 890 (79.5); 0.001 5 (71.4); >0.99	56 (59.6); <0.0001 24 (51.1); 0.0001 832 (74.3); <0.0001 5 (71.4); >0.99
HER2 status			
Negative (n = 1127) Positive (n = 130) Unknown (n = 11)	959 (85.1); ref. 118 (90.8); ref. 9 (81.8); ref.	869 (77.1); <0.0001 108 (83.1); 0.09 6 (54.6); 0.38	809 (71.8); <0.0001 102 (78.5); 0.005 6 (54.6); 0.38
Interval cancers (n = 562)*			
Tumour size			
0–10 mm (n = 90) 11–20 mm (n = 216) 21–50 mm (n = 201) 51 + mm (n = 35) Unknown (n = 20)	9 (10.0); ref. 11 (5.1); ref. 13 (6.5); ref. 6 (17.1); ref. 0 (0.0); ref.	23 (25.6); 0.004 48 (22.2); <0.0001 52 (25.9); <0.0001 13 (37.1); 0.04 3 (15.0); <0.0001^†^	17 (18.9); 0.12 39 (18.1); <0.0001 40 (19.9); <0.0001 10 (28.6); 0.22 3 (15.0); <0.0001^†^
Malignancy grade			
Grade 1 (n = 104) Grade 2 (n = 239) Grade 3 (n = 166) Unknown (n = 53)	8 (7.7); ref. 15 (6.3); ref. 13 (7.8); ref. 3 (5.7); ref.	24 (23.1); 0.003 77 (32.2); <0.0001 32 (19.3); 0.001 6 (11.3); 0.45	21 (20.2); 0.01 60 (25.1); <0.0001 24 (14.5); 0.04 4 (7.6); >0.99
TNM stage			
Local (I + II) (n = 508) Locally advanced (III) (n = 33) Distant metastasis (IV) (n = 16) Unknown (n = 5)	34 (6.7); ref. 4 (12.1); ref. 0 (0.0); ref. 1 (20.0); ref.	129 (25.4); <0.0001 6 (18.2); 0.69 3 (18.8); <0.0001^†^ 1 (20.0); >0.99^†^	100 (19.7); <0.0001 5 (15.2); >0.99 3 (18.8); <0.0001^†^ 1 (20.0); >0.99^†^
Lymph node positivity			
No (n = 376) Yes (n = 186)	26 (6.9); ref. 13 (7.0); ref.	83 (22.1); <0.0001 56 (30.1); <0.0001	67 (17.8); <0.0001 42 (22.6); <0.0001
ER positivity			
0% (n = 113) 1–9% (n = 51) 10–100% (n = 394) Unknown (n = 4)	8 (7.1); ref. 2 (3.9); ref. 29 (7.4); ref. 0 (0.0); ref.	15 (13.3); 0.07 10 (19.6); 0.02 113 (28.7); <0.0001 1 (25.0); <0.0001^†^	11 (9.7); 0.58 9 (17.7); 0.04 88 (22.3); <0.0001 1 (25.0) < 0.0001^†^
HER2 status			
Negative (n = 454) Positive (n = 95) Unknown (n = 13)	33 (7.3); ref. 5 (5.3); ref. 1 (7.7); ref.	117 (25.8); <0.0001 20 (21.1); 0.0003 2 (15.4); 0.26^†^	93 (20.5); <0.0001 14 (14.7); 0.04 2 (15.4); 0.26^†^

Data are n (%); *p* value. The cancer detection rate is reported as the number of detected cancers out of the number of true cancers for the subgroup in the same row. TNM = tumour, node, metastasis. ER = estrogen receptor. HER2 = human epidermal growth factor receptor 2. AI_sens_=artificial intelligence score cut-off point matched at mean first reader sensitivity. AI_spec_=artificial intelligence score cut-off point matched at mean first reader specificity. *Reported for invasive cancers only. †Exact binomial test used instead of McNemar’s test due to small discordant cells

### AI-integrated screening accuracy

Integrated AI_sens_ achieved a higher sensitivity by + 2.3% (*p* = 0.0004) compared to combined reading, at the cost of a lower specificity (-0.6%) and PPV (-3.9%), and higher recall rate (+ 0.6%) and arbitration rate (+ 2.2%) (*p* < 0.0001 for all). In absolute terms, this corresponded to 1708 recalls (+ 24.9%) and 5831 arbitrations (+ 78.4%) (Supplementary eTable 4). Integrated AI_spec_ showed no significant difference in any of the outcome measures apart from a higher arbitration rate by + 1.1% (*p* < 0.0001), amounting to 2841 (+ 38.2%) arbitrations (Supplementary eTable 4). Compared to the combined reading, detection rates in relation to screen-detected cancers were lower for Integrated AI_sens_ by 54 (-3.7%) cancers and for Integrated AI_spec_ by 66 (-4.5%) cancers but were higher in relation to interval cancers by 100 (+ 17.8%) cancers and 79 (+ 14.1%) cancers, respectively (*p* < 0.0001 for all) (Table 3). Subgroup analyses showed a lower proportion of detection discrepancies compared to the Standalone AI scenario, with only few interval cancers being missed in the AI-integrated screening and detected by the combined reading, and no screen-detected cancers being missed by the combined reading (Supplementary eTable 3).

### Next-round screen-detected and long-term cancers

When including next-round screen-detected cancers and long-term cancers in the accuracy analysis, the sensitivity of Standalone AI and Integrated AI with both thresholds were statistically significantly higher than first reader and combined reading, respectively (*p* < 0.0001 for all), with varying statistically significantly lower, higher, or no different specificity (Supplementary eTable 5). However, the sensitivity of the index test and comparator were notably lower compared to those presented in Table 2.

## Discussion

### Summary of findings

We achieved a large representative study sample with a cancer detection rate and recall rate in line with previous reports on screening outcome from Danish screening rounds [4, 19]. In the Standalone AI scenario, the accuracy at both AI abnormality thresholds was found statistically significantly lower than that of the first reader across most outcome measures, mainly due to lower detection of scree-detected cancers. However, the AI system had a statistically significantly higher interval cancer detection rate and a higher accuracy across most outcome measures when next-round screen-detected cancers and long-term cancers were included in the cancer outcome. In the AI-integrated screening scenario, detection accuracy was at the level of or statistically significantly higher than the combined reading, depending on the chosen threshold, only with a slightly higher arbitration rate. A statistically significantly higher recall rate was observed for Integrated AI_sens_ but not for Integrated AI_spec_. A notable proportion of cancers were missed by the AI system and detected by first reader, and vice versa, although detection discrepancies were to a lesser extent evident in the AI-integrated screening scenario.

### Comparison with literature

Our results on Standalone AI accuracy corroborate findings observed by Leibig and colleagues who reported significantly lower sensitivity and specificity of an in-house and commercial AI system in a standalone AI pathway compared to a single unaided radiologist, when the threshold was set to maintain the radiologist’s sensitivity [20]. Schaffter and colleagues showed significantly lower specificity by both an in-house top-performing AI system and an aggregated ensemble of top-performing AI algorithms compared to first reader and consensus reading, when sensitivity was set to match that of first reader [21]. Conversely, multiple other studies reported equal or higher standalone AI accuracy compared to human readers [10–12, 22], however, most had overall high risk of bias or applicability concerns according to several systematic reviews [13, 23, 24]. Numerous studies have explored different simulated screening scenarios with an AI system, for instance as reader aid or triage, and although many report higher AI accuracy, these also suffer from similar methodological limitations [13, 23, 24].

Among the possible implementation strategies within double reading, partial replacement with AI replacing one reader seems to be the preferred AI-integrated screening scenario by breast screening readers [25], although only few recent studies, other than the current, have investigated this scenario. Larsen and colleagues evaluated the same AI system tested in this study as one of two readers in a setting in which abnormal readings were sent to consensus [26]. Using different consensus selection thresholds in two scenarios yielded a lower recall rate, higher consensus rate, and overall higher sensitivity when including interval cancer. However, AI-selected cases for consensus, missing an original consensus decision in the dataset, were not included in the decision outcome of the scenarios, creating uncertainty around the reliability of the recall and accuracy estimates. Sharma and colleagues tested an in-house commercial AI system in a simulated double reading with AI as one reader, which showed non-inferiority or superiority across all accuracy metrics compared to non-blinded double reading with arbitration, although the arbitration rate was not reported [27]. The study used historical second reader decisions as arbitration outcomes in cases where the original arbitration was absent, meaning that the AI decision was not included in the comparison, which could have caused an underestimation of the differences in accuracy between the AI and the radiologists. An unpublished study by Frazer and colleagues evaluated an in-house AI system in a reader-replacement scenario in which the arbitration outcome for a missing historic arbitration was simulated by matching the retrospective third-reading performance, as in the current study [28]. Compared to double reading with arbitration, the AI-integrated screening scenario with the improved system threshold achieved higher sensitivity and specificity and a lower recall rate at the cost of a highly increased arbitration rate. Unfortunately, > 25% of the study population was excluded, mostly due to lack of follow-up, introducing a high risk of selection bias.

### Methodological considerations and limitations

In addition to many studies lacking a representative study sample, comparison of results across the literature is further complicated by varying choice of comparators, reference standard, abnormality threshold levels, and inconsistency in applying accuracy measures in accordance to reporting guidelines [13, 29]. Contrary to previous research, the main strengths of this study were the unselected, consecutive population-wide cohort, availability of high-quality follow-up data with a low exclusion rate, and subspecialised breast radiologists as comparators, thereby representing a more reliable real-life population and reference standard. By simulating the arbitration decision to match the arbitrator’s accuracy, when original arbitrations were absent, we could achieve more realistic estimates of the accuracy outcomes in the AI-integrated screening scenario, although this did not take into account how AI implementation can alter radiologists’ behaviour or decisions in a clinical setting. It should be stressed that standalone applications of AI, as evaluated in this study, are for now not clinically possible nor justified due to legal and ethical limitations among others.

Our work did have several limitations. The chosen AI score cut-off points were derived based on the sample in the current study which could lead to loss of generalisability to other screening populations with a differing screening setting and workflow, diverse ethnic groups, and imaging vendors among others. For instance, the image data in the study were derived from only one mammography vendor, limiting the generalisability of results to mammograms acquired from other sources. Hence, differences or changes in a screening site’s technical setup or other factors affecting image output should be considered when deciding on a relevant AI threshold in relation to AI deployment in clinical practice. This could prospectively be resolved by having a local validation dataset or procedure in case of any such changes or variations in external or internal factors related to the AI system, through which a site-based adaptive strategy for threshold selection can be devised.

Most other limitations were related to the retrospective nature of this study, among which is the lack of diagnostic work-up on cases recalled by the AI system but not by radiologists. If these were true positive but not detected within the same screening round, the accuracy of the AI system would be underestimated. Conversely, recalls of cases without cancer at screening but with an interval cancer developing before the next round would count as true positives, and since exact AI cancer-suspected areas were not evaluated for false positive markings, AI accuracy could have been overestimated. Hence, abnormal AI predictions could be clinically significant cancers, overdiagnosed cancers, or false positives. The magnitude of such potential prediction misclassifications and thereby bias skewing the accuracy estimates is difficult to assess in mammography screening without a gold standard for all participants, such as MRI or other imaging along with biopsy, as it would be unnecessary and unethical to subject all women to comprehensive testing. Our findings of a higher detection rate of interval cancers and higher accuracy in both scenarios, when including next-round screen-detected and long-term cancers (Supplementary eTable 5), could indicate a tendency towards an underestimation of AI accuracy due to the current definition of the reference standard and the lack of a gold standard in mammography screening. However, the number of true positive AI-detected cancers might be limited in view of findings in a previous study showing that only 58% of AI-marked interval cancers, which were considered missed by radiologists or had minimal radiographic malignancy signs (i.e. false negatives), were correctly located and could potentially be detected at screening [30]. This study used an older version of the same AI system as the current study but at a threshold score of 9.01 compared to 9.57 and 9.71 for AI_sens_ and AI_spec_, respectively. Furthermore, the majority of interval cancers have been reported to be comprised of true or occult interval cancers [31], which even with AI-prompts would not be expected to be detected at screening or diagnostic work-up. These findings relating to interval cancers should not be less valid for next-round screen-detected and long-term cancers, and in particular cancers with a short doubling time, such as grade 3 tumours, making it unlikely for these to have been detected with an AI positive assessment. The reported results on interval cancers which were missed by human readers but detected by or with the AI system (Supplementary eTables 2–3), especially those diagnosed ≥ 12 months after screening, should therefore be interpreted with caution in light of the radiological and biological characteristics of interval cancers.

What further contributes to the uncertainty around estimates in accuracy studies of this type is the intrinsic verification bias due to different reference standards depending on the screening decision outcome [32]. The choice of management to confirm disease status was, for instance, correlated with the readers’ screen decisions, likely introducing a systematic bias favouring the accuracy of the radiologists.

While our study design reinforces the reliability and generalisability of the findings in this study, we recognise that more accurate quantification of the actual detection accuracy of AI requires prospective studies which have the advantage of estimating the effect of AI-integrated screening on detection accuracy and workload. This is further emphasised considering that the workload reduction achieved in this study for Integrated AI_sens_ through decreasing human screen reads with > 48% would to some degree be counterbalanced by the found increase in recall rate of almost 25% (Supplementary eTable 4). Only with Integrated AI_spec_, which showed a stable recall rate, AI-integrated screening could be considered feasible enough to ensure actual alleviation of workforce pressures, stressing the importance of selecting an appropriate AI threshold value. Well-designed randomised controlled trials are warranted to elucidate the implications of clinical implementation of AI as one of two readers in mammography screening, the choice of a clinically relevant threshold, as well as the effects on cancer detection, workflow, and radiologist interpretation and behaviour. The first two prospective studies reported only recently short-term results of population-based AI-integrated screening with positive screening outcome in terms of cancer detection rate and workload reduction, providing a promising outlook for safe AI deployment within mammography screening [33, 34].

## Conclusions

In conclusion, findings of this retrospective and population-wide mammography screening accuracy study suggest that an AI system with an appropriate threshold could be feasible as a replacement of the first reader in double reading with arbitration. The spectrum of detected cancers differed significantly across multiple cancer subgroups with a general tendency of lower accuracy for screen-detected cancers and higher accuracy for interval cancers. Discrepancies in cancers detected by the AI system and radiologists could be harnessed to improve detection accuracy of particular subtypes of interval cancers by applying AI for decision support in double reading.

### Electronic supplementary material

Below is the link to the electronic supplementary material.

Supplementary Material 1

## Data Availability

The image dataset and local radiology dataset collected for this study are not publicly available due to Danish and European regulations for the use of data in research, but investigators are on reasonable request encouraged to contact the corresponding author for academic inquiries into the possibility of applying for deidentified participant data through a Data Transfer Agreement procedure. The registry datasets from the Danish Breast Cancer Cooperative Group (DBCG) and the Danish Quality Database on Mammography Screening (DKMS) used and analysed during the current study are available from the Danish Clinical Quality Program – National Clinical Registries (RKKP), but restrictions apply to the availability of these data, which were used under license for the current study, and so are not publicly available. Data are, however, available upon reasonable request to and with permission from the RKKP, provided that the necessary access requirements are fulfilled.

## Abbreviations

AI: Artificial intelligence
AI_sens_: Artificial intelligence score cut-off point matched at mean first reader sensitivity
AI_spec_: Artificial intelligence score cut-off point matched at mean first reader specificity
CE: Conformité Europëenne
CI: Confidence interval
DCIS: Ductal carcinoma in situ
DICOM: Digital Imaging and Communications in Medicine
FDA: Food and Drug Administration
NPV: Negative predictive value
PPV: Positive predictive value
RSD: Region of Southern Denmark
SD: Standard deviation
STARD: Standards for Reporting Diagnostic Accuracy Studies
