# Supplementary material for: Breast cancer detection accuracy of AI in an entire screening population: a retrospective, multicentre study
Source: Cancer Imaging. 2023 Dec 20;23:127. doi: 10.1186/s40644-023-00643-x (PMC10731688; doi:10.1186/s40644-023-00643-x)
Supplement: Supplementary file 1 — Supplementary Material 1 [file 40644_2023_643_MOESM1_ESM.pdf]

Supplementary Appendix materials for

Breast cancer detection accuracy of AI in an entire screening population: a retrospective, multicentre study

Mohammad Talal Elhakim, MD[1,2]; Sarah Wordenskjold Stougaard MSc[2]; Ole Graumann, PhD[1,3,4]; Mads Nielsen, PhD [5]; Kristina Lång, PhD[6,7]; Oke Gerke, PhD[2,8]; Lisbet Brønsro Larsen, MD[1]; Benjamin Schnack Brandt Rasmussen, PhD[1,2,9]

[1] Department of Radiology, Odense University Hospital, Kløvervænget 47, Entrance 27, Ground floor, 5000 Odense C, Denmark
[2] Department of Clinical Research, University of Southern Denmark, Kløvervænget 10, Entrance 112, 2nd floor, 5000 Odense C, Denmark
[3] Department of Radiology, Aarhus University Hospital, Palle Juul-Jensens Blvd. 99, 8200 Aarhus N, Denmark
[4] Department of Clinical Research, Aarhus University, Palle Juul-Jensens Blvd. 99, 8200 Aarhus N, Denmark
[5] Department of Computer Science, University of Copenhagen, Universitetsparken 1, 2100 København Ø, Denmark
[6] Department of Translational Medicine, Lund University, Inga Maria Nilssons gata 47, SE-20502, Malmö, Sweden
[7] Unilabs Mammography Unit, Skåne University Hospital, Jan Waldenströms gata 22, SE-20502, Malmö, Sweden
[8] Department of Nuclear Medicine, Odense University Hospital, Kløvervænget 47, Entrance 44, 5000 Odense C, Denmark
[9] CAI-X – Centre for Clinical Artificial Intelligence, Odense University Hospital, Kløvervænget 8C, Entrance 102, 5000 Odense C, Denmark

Corresponding author:
Name: Mohammad Talal Elhakim, MD
Address: Kløvervænget 10, Entrance 112, 2nd floor, 5000 Odense C, Denmark
Email: mte@rsyd.dk

Contents

Supplementary eMethod 1. Standard for Reporting of Diagnostic Accuracy Studies (STARD) checklist p 2

Supplementary eFigure 1. Distribution of Transpara exam scores across the study sample p 3

Supplementary eTable 1. Detection accuracy analysis across radiologist position p 4

Supplementary eTable 2. Detection agreements and discrepancies across cancer subgroups in the Standalone AI scenario p 5

Supplementary eTable 3. Detection agreements and discrepancies across cancer subgroups in the AI-integrated screening scenario p 6

Supplementary eTable 4. Comparison of screening outcome and results of the reference standard in both study scenarios with descriptive workload analysis p 7

Supplementary eTable 5. Detection accuracy analysis with inclusion of next-round screen-detected cancers and long-term cancers in both study scenarios p 8

Supplementary eMethod 1: Standard for Reporting of Diagnostic Accuracy Studies (STARD) checklist

Section & Topic	No	Item	Reported on page # (submitted version)
**TITLE OR ABSTRACT**			
	**1**	Identification as a study of diagnostic accuracy using at least one measure of accuracy (such as sensitivity, specificity, predictive values, or AUC)	1-2
**ABSTRACT**			
	**2**	Structured summary of study design, methods, results, and conclusions (for specific guidance, see STARD for Abstracts)	2
**INTRODUCTION**			
	**3**	Scientific and clinical background, including the intended use and clinical role of the index test	3
	**4**	Study objectives and hypotheses	3
**METHODS**			
Study design	**5**	Whether data collection was planned before the index test and reference standard were performed (prospective study) or after (retrospective study)	4
Participants	**6**	Eligibility criteria	4
	**7**	On what basis potentially eligible participants were identified (such as symptoms, results from previous tests, inclusion in registry)	4
	**8**	Where and when potentially eligible participants were identified (setting, location and dates)	4
	**9**	Whether participants formed a consecutive, random or convenience series	4
Test methods	**10a**	Index test, in sufficient detail to allow replication	5-6
	**10b**	Reference standard, in sufficient detail to allow replication	5-6
	**11**	Rationale for choosing the reference standard (if alternatives exist)	5-6
	**12a**	Definition of and rationale for test positivity cut-offs or result categories of the index test, distinguishing pre-specified from exploratory	5-6
	**12b**	Definition of and rationale for test positivity cut-offs or result categories of the reference standard, distinguishing pre-specified from exploratory	4-6
	**13a**	Whether clinical information and reference standard results were available to the performers/readers of the index test	-
	**13b**	Whether clinical information and index test results were available to the assessors of the reference standard	-
Analysis	**14**	Methods for estimating or comparing measures of diagnostic accuracy	6-7
	**15**	How indeterminate index test or reference standard results were handled	4
	**16**	How missing data on the index test and reference standard were handled	4
	**17**	Any analyses of variability in diagnostic accuracy, distinguishing pre-specified from exploratory	6-7
	**18**	Intended sample size and how it was determined	-
**RESULTS**			
Participants	**19**	Flow of participants, using a diagram	8, Figure 2
	**20**	Baseline demographic and clinical characteristics of participants	8, Table 1
	**21a**	Distribution of severity of disease in those with the target condition	8, Tables 1,3,4
	**21b**	Distribution of alternative diagnoses in those without the target condition	-
	**22**	Time interval and any clinical interventions between index test and reference standard	-
Test results	**23**	Cross tabulation of the index test results (or their distribution) by the results of the reference standard	9, eTable 4
	**24**	Estimates of diagnostic accuracy and their precision (such as 95% confidence intervals)	9, Table 2, eTable 5
	**25**	Any adverse events from performing the index test or the reference standard	-
**DISCUSSION**			
	**26**	Study limitations, including sources of potential bias, statistical uncertainty, and generalisability	16-18
	**27**	Implications for practice, including the intended use and clinical role of the index test	15-18
**OTHER INFORMATION**			
	**28**	Registration number and name of registry	4,19
	**29**	Where the full study protocol can be accessed	-
	**30**	Sources of funding and other support; role of funders	20

Supplementary eFigure 1: Distribution of Transpara exam scores across the study sample

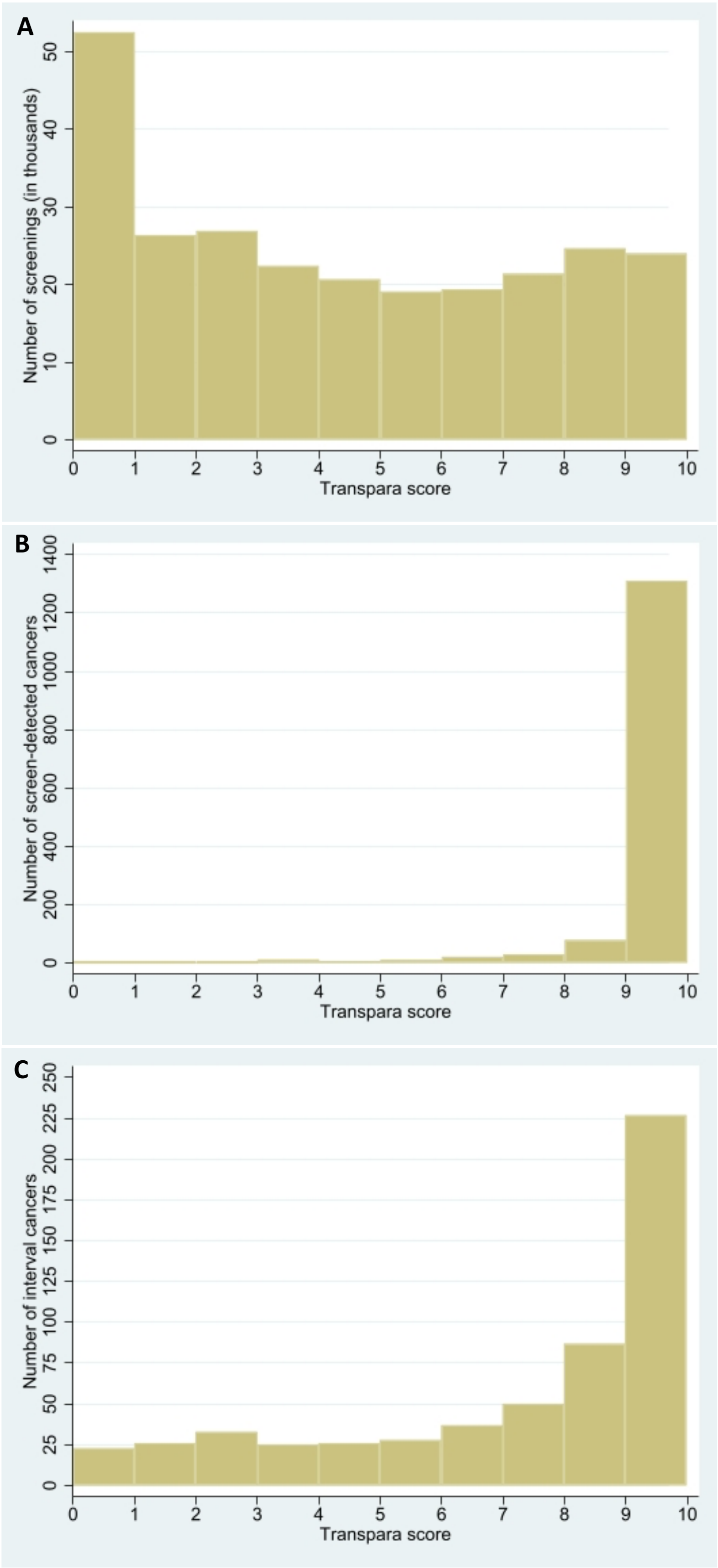

(A) The Transpara exam score distributed across (A) all screening mammograms, (B) screen-detected cancers, and (C) interval cancers in the study sample. The Transpara exam score was precalibrated in such a way that the number of screening mammograms in each category should be almost equal, i.e., 10% of the mammograms would fall into each category of scores. When matching the AI score thresholds in the study to the mean sensitivity and mean specificity of the first reader, AI_{sens} and AI_{spec} used a Transpara exam score of 9.56858 and 9.70159, respectively.

Supplementary eTable 1: Detection accuracy analysis across radiologist position

	Sensitivity (95% CI); p value*	Specificity (95% CI); p value*	PPV (95% CI); p value†	NPV (95% CI); p value†	Recall rate (95% CI); p value†
First reader	63.7 (61.6-65.8); ref.	97.8 (97.7-97.8); ref.	18.7 (17.8-19.6); ref.	99.7 (99.7-99.7); ref.	2.7 (2.6-2.8); ref.
Second reader	68.3 (66.2-70.3); <0.0001	98.0 (98.0-98.1); <0.0001	21.7 (20.7-22.7); <0.0001	99.7 (99.7-99.8); 0.004	2.5 (2.4-2.6); <0.0001
Arbitrator	90.8 (87.5-93.5); <0.0001	53.0 (51.8-54.1); <0.0001	9.7 (8.7-10.7); <0.0001	99.0 (98.7-99.7); <0.0001	49.3 (48.2-50.5); <0.0001
Combined reading	73.9 (72.0-75.8); <0.0001	97.9 (97.9-98.0); <0.0001	22.0 (21.0-23.0); <0.0001	99.8 (99.8-99.8); <0.0001	2.7 (2.6-2.7); 0.18

Data are % (95% CI); p value. PPV=positive predictive value. NPV=negative predictive value. *p values were calculated using McNemar's test. †p values were calculated using exact binomial test.

Supplementary eTable 2: Detection agreements and discrepancies across cancer subgroups in the Standalone AI scenario

	Detected by both First reader and		Detected by First reader, missed by		Missed by First reader, detected by		Missed by both First reader and	
	Standalone AI_{sens}	Standalone AI_{spec}	Standalone AI_{sens}	Standalone AI_{spec}	Standalone AI_{sens}	Standalone AI_{spec}	Standalone AI_{sens}	Standalone AI_{spec}
**All cancers (n=2041)**	1043 (51.1)	981 (48.1)	258 (12.6)	320 (15.7)	258 (12.6)	216 (10.6)	482 (23.6)	254 (25.7)
**Screen-detected cancers (n=1479)**	1018 (68.8)	961 (65.0)	244 (16.5)	301 (20.4)	144 (9.7)	127 (8.6)	73 (4.9)	90 (6.1)
**Interval cancers (n=562)**	25 (4.4)	20 (3.6)	14 (2.5)	19 (3.4)	114 (20.3)	89 (15.8)	409 (72.8)	434 (77.2)
<12 months after screening (n=170)	7 (4.1)	6 (3.5)	6 (3.5)	7 (4.1)	36 (21.2)	30 (17.6)	121 (71.2)	127 (74.7)
≥12 months after screening (n=392)	18 (4.6)	14 (3.6)	8 (2.0)	12 (3.1)	78 (19.9)	59 (15.1)	288 (73.5)	307 (78.3)
**Histological subtype**								
Invasive ductal (n=1393)	733 (52.6)	689 (49.5)	172 (12.3)	216 (15.5)	174 (12.5)	144 (10.3)	314 (22.5)	344 (24.7)
Invasive lobular (n=222)	93 (41.9)	87 (39.2)	24 (10.8)	30 (13.5)	35 (15.8)	27 (12.2)	70 (31.5)	78 (35.1)
Other invasive (n=215)	65 (30.2)	60 (27.9)	38 (17.7)	43 (20.0)	22 (10.2)	19 (8.8)	90 (41.9)	93 (43.3)
Ductal carcinoma in situ (n=211)	152 (72.0)	145 (68.7)	24 (11.4)	31 (14.7)	27 (12.8)	26 (12.3)	8 (3.8)	9 (4.3)
**Tumour size***								
0-10 mm (n=577)	302 (52.3)	277 (48.0)	97 (16.8)	122 (21.1)	77 (13.3)	65 (11.3)	101 (17.5)	113 (19.6)
11-20 mm (n=790)	431 (54.6)	409 (51.8)	90 (11.4)	112 (14.2)	82 (10.4)	66 (8.4)	187 (23.7)	203 (25.7)
21-50 mm (n=380)	136 (35.8)	129 (33.9)	38 (10.0)	45 (11.8)	58 (15.3)	48 (12.6)	148 (38.9)	158 (41.6)
51+ mm (n=49)	15 (30.6)	15 (30.6)	2 (4.1)	2 (4.1)	11 (22.4)	8 (16.3)	21 (42.9)	24 (49.0)
Unknown (n=34)	7 (20.6)	6 (17.6)	7 (20.6)	8 (23.5)	3 (8.8)	3 (8.8)	17 (50.0)	17 (50.0)
**Malignancy grade***								
Grade 1 (n=507)	283 (55.8)	263 (51.9)	48 (9.5)	68 (13.4)	76 (15.0)	61 (12.0)	100 (19.7)	115 (22.7)
Grade 2 (n=815)	417 (51.2)	395 (48.5)	103 (12.6)	125 (15.3)	109 (13.4)	92 (11.3)	186 (22.8)	203 (24.9)
Grade 3 (n=358)	141 (39.4)	131 (36.6)	52 (14.5)	62 (17.3)	33 (9.2)	26 (7.3)	132 (36.9)	139 (38.8)
Unknown (n=150)	50 (33.3)	47 (31.3)	31 (20.7)	34 (22.7)	13 (8.7)	11 (7.3)	56 (37.3)	58 (38.7)
**TNM stage***								
Local (I + II) (n=1761)	876 (49.7)	822 (46.7)	229 (13.0)	283 (16.1)	224 (12.7)	184 (10.4)	432 (24.5)	472 (26.8)
Locally advanced (III) (n=44)	10 (22.7)	10 (22.7)	5 (11.4)	5 (11.4)	4 (9.1)	3 (6.8)	25 (56.8)	26 (59.1)
Distant metastasis (IV) (n=20)	4 (20.0)	3 (15.0)	0 (0.0)	1 (5.0)	3 (15.0)	3 (15.0)	13 (65.0)	13 (65.0)
Unknown (n=5)	1 (20.0)	1 (20.0)	0 (0.0)	0 (0.0)	0 (0.0)	0 (0.0)	4 (80.0)	4 (80.0)
**Lymph node positivity***								
No (n=1340)	665 (49.6)	624 (46.6)	175 (13.1)	216 (16.1)	161 (12.0)	135 (10.1)	339 (25.3)	365 (27.2)
Yes (n=490)	226 (46.1)	212 (43.3)	59 (12.0)	73 (14.9)	70 (14.3)	55 (11.2)	135 (27.6)	150 (30.6)
**ER positivity***								
0% (n=207)	62 (30.0)	57 (27.5)	34 (16.4)	39 (18.8)	13 (6.3)	10 (4.8)	98 (47.3)	101 (48.8)
1-9% (n=98)	26 (26.5)	22 (22.4)	20 (20.4)	24 (24.5)	12 (12.2)	11 (11.2)	40 (40.8)	41 (41.8)
10-100% (n=1514)	799 (52.8)	753 (49.7)	178 (11.8)	224 (14.8)	204 (13.5)	167 (11.0)	333 (22.0)	370 (24.4)
Unknown (n=11)	4 (36.4)	4 (35.4)	2 (18.2)	2 (18.2)	2 (18.2)	2 (18.2)	3 (27.3)	3 (27.3)
**HER2 status***								
Negative (n=1581)	782 (49.5)	733 (46.4)	210 (13.3)	259 (16.4)	204 (12.9)	169 (10.7)	385 (24.4)	420 (26.6)
Positive (n=225)	103 (45.8)	97 (43.1)	20 (8.9)	26 (11.6)	25 (11.1)	19 (8.4)	77 (34.2)	83 (36.9)
Unknown (n=24)	6 (25.0)	5 (25.0)	4 (16.7)	4 (16.7)	2 (8.3)	2 (8.3)	12 (50.0)	12 (50.0)

Data are n (%). The cancer detection rate is reported as the number of detected cancers out of the number of true cancers for the subgroup in the same row.TNM=tumour, node, metastasis. ER=estrogen receptor. HER2=human epidermal growth factor receptor 2. AI_{sens}=artificial intelligence score cut-off point matched at mean first reader sensitivity. AI_{spec}=artificial intelligence score cut-off point matched at mean first reader specificity. *Reported for invasive cancers only.

Supplementary eTable 3: Detection agreements and discrepancies across cancer subgroups in the AI-integrated screening scenario

	Detected by both Combined reading and		Detected by Combined reading, missed by		Missed by Combined reading, detected by		Missed by both Combined reading and	
	Integrated AI_{sens}	Integrated AI_{spec}	Integrated AI_{sens}	Integrated AI_{spec}	Integrated AI_{sens}	Integrated AI_{spec}	Integrated AI_{sens}	Integrated AI_{spec}
**All cancers (n=2041)**	1450 (71.0)	1439 (70.5)	59 (2.9)	70 (3.4)	105 (5.1)	83 (4.1)	427 (20.9)	449 (22.0)
**Screen-detected cancers (n=1479)**	1425 (96.3)	1413 (95.5)	54 (3.7)	66 (4.5)	0 (0.0)	0 (0.0)	0 (0.0)	0 (0.0)
**Interval cancers (n=562)**	27 (4.8)	27 (4.8)	3 (0.5)	3 (0.5)	103 (18.3)	82 (14.6)	429 (76.3)	450 (80.1)
<12 months after screening (n=170)	13 (7.6)	13 (7.6)	1 (0.6)	1 (0.6)	34 (20.0)	28 (16.5)	122 (71.8)	128 (75.3)
≥12 months after screening (n=392)	14 (3.6)	14 (3.6)	2 (0.5)	2 (0.5)	69 (17.6)	54 (13.8)	307 (78.3)	322 (82.1)
**Histological subtype**								
Invasive ductal (n=1393)	996 (71.5)	990 (71.1)	38 (2.7)	44 (3.2)	76 (5.5)	63 (4.5)	283 (20.3)	296 (21.2)
Invasive lobular (n=222)	137 (61.7)	134 (60.4)	6 (2.7)	9 (4.1)	17 (7.7)	11 (5.0)	62 (27.9)	68 (30.6)
Other invasive (n=215)	110 (51.2)	113 (52.6)	11 (5.1)	8 (3.7)	12 (5.6)	9 (4.2)	82 (38.1)	85 (39.5)
Ductal carcinoma in situ (n=211)	207 (98.1)	202 (95.7)	4 (1.9)	9 (4.3)	0 (0.0)	0 (0.0)	0 (0.0)	0 (0.0)
**Tumour size***								
0-10 mm (n=577)	468 (81.1)	469 (81.3)	28 (4.9)	27 (4.7)	16 (2.8)	13 (2.3)	65 (11.3)	68 (11.8)
11-20 mm (n=790)	560 (70.9)	554 (70.1)	21 (2.7)	27 (3.4)	38 (4.8)	29 (3.7)	171 (21.6)	180 (22.8)
21-50 mm (n=380)	185 (48.7)	183 (48.2)	4 (1.1)	6 (1.6)	41 (10.8)	33 (8.7)	150 (39.5)	158 (41.6)
51+ mm (n=49)	17 (34.7)	17 (34.7)	1 (2.0)	1 (2.0)	7 (14.3)	5 (10.2)	24 (49.0)	26 (53.1)
Unknown (n=34)	13 (38.2)	14 (41.2)	1 (2.9)	0 (0.0)	3 (8.8)	3 (8.8)	17 (50.0)	17 (50.0)
**Malignancy grade***								
Grade 1 (n=507)	91 (60.7)	94 (62.7)	8 (5.3)	5 (3.3)	5 (3.3)	3 (2.0)	46 (30.7)	48 (32.0)
Grade 2 (n=815)	397 (78.3)	391 (77.1)	13 (2.6)	19 (3.7)	21 (4.1)	19 (3.7)	76 (15.0)	78 (15.4)
Grade 3 (n=358)	561 (68.8)	560 (68.7)	26 (3.2)	27 (3.3)	56 (6.9)	45 (5.5)	172 (21.1)	183 (22.5)
Unknown (n=150)	194 (54.2)	192 (53.6)	8 (2.2)	10 (2.8)	23 (6.4)	16 (4.5)	133 (37.2)	140 (39.1)
**TNM stage***								
Local (I + II) (n=1761)	1226 (69.6)	1219 (69.2)	54 (3.1)	61 (3.5)	98 (5.6)	77 (4.4)	383 (21.7)	404 (22.9)
Locally advanced (III) (n=44)	12 (27.3)	13 (29.5)	1 (2.3)	0 (0.0)	4 (9.1)	3 (6.8)	27 (61.4)	28 (63.6)
Distant metastasis (IV) (n=20)	4 (20.0)	4 (20.0)	0 (0.0)	0 (0.0)	3 (15.0)	3 (15.0)	13 (65.0)	13 (65.0)
Unknown (n=5)	1 (20.0)	1 (20.0)	0 (0.0)	0 (0.0)	0 (0.0)	0 (0.0)	4 (80.0)	4 (80.0)
**Lymph node positivity***								
No (n=1340)	942 (70.3)	941 (70.2)	42 (3.1)	43 (3.2)	63 (4.7)	51 (3.8)	293 (21.9)	305 (22.8)
Yes (n=490)	301 (61.4)	296 (60.4)	13 (2.7)	18 (3.7)	42 (8.6)	32 (6.5)	134 (27.3)	144 (29.4)
**ER positivity***								
0% (n=207)	91 (44.0)	94 (45.4)	11 (5.3)	8 (3.9)	9 (4.3)	7 (3.4)	96 (46.4)	98 (47.3)
1-9% (n=98)	48 (49.0)	45 (45.9)	1 (1.0)	4 (4.1)	9 (9.2)	6 (6.1)	40 (40.8)	43 (43.9)
10-100% (n=1514)	1097 (72.5)	1091 (72.1)	43 (2.8)	49 (3.2)	86 (5.7)	69 (4.6)	288 (19.0)	305 (20.1)
Unknown (n=11)	7 (63.6)	7 (63.6)	0 (0.0)	0 (0.0)	1 (9.1)	1 (9.1)	3 (27.3)	3 (27.3)
**HER2 status***								
Negative (n=1581)	1105 (69.9)	1097 (69.4)	46 (2.9)	54 (3.4)	89 (5.6)	71 (4.5)	341 (21.6)	359 (22.7)
Positive (n=225)	127 (56.4)	129 (57.3)	8 (3.6)	6 (2.7)	15 (6.7)	11 (4.9)	75 (33.3)	79 (35.1)
Unknown (n=24)	11 (45.8)	11 (45.8)	1 (4.2)	1 (4.2)	1 (4.2)	1 (4.2)	11 (45.8)	11 (45.8)

Data are n (%). The cancer detection rate is reported as the number of detected cancers out of the number of true cancers for the subgroup in the same row.TNM=tumour, node, metastasis. ER=estrogen receptor. HER2=human epidermal growth factor receptor 2. AI_{sens}=artificial intelligence score cut-off point matched at mean first reader sensitivity. AI_{spec}=artificial intelligence score cut-off point matched at mean first reader specificity. *Reported for invasive cancers only.

Supplementary eTable 4: Comparison of screening outcome and results of the reference standard in both study scenarios with descriptive workload analysis.

	Standalone AI			AI-integrated screening		
	**First reader**	**Standalone AI_{sens}**	**Standalone AI_{spec}**	**Combined reading**	**Integrated AI_{sens}**	**Integrated AI_{spec}**
**Number of episodes**						
True positive	1301 (ref.)	1301 (0.0)	1197 (-7.9)	1509 (ref.)	1555 (+3.0)	1522 (+0.9)
True negative	249959 (ref.)	246590 (-1.3)	249952 (<-0.1)	250278 (ref.)	248616 (-0.7)	250234 (<-0.1)
False positive	5671 (ref.)	9040 (+59.4)	5678 (+0.1)	5352 (ref.)	7014 (+31.1)	5396 (+0.8)
False negative	740 (ref.)	740 (0.0)	844 (+14.1)	532 (ref.)	486 (-8.6)	519 (-2.4)
**Workload**						
Recalls	6972 (ref.)	10341 (+48.3)	6875 (-1.4)	6861 (ref.)	8569 (+24.9)	6918 (+0.8)
Human readings	257671 (ref.)	0 (-100)	0 (-100)	522105 (ref.)	270936 (-48.1)	267946 (-48.7)
Arbitrations	NA	NA	NA	7434 (ref.)	13265 (+74.4)	10275 (+38.2)

Data are n (Δ%). AI_{sens}=artificial intelligence score cut-off point matched at mean first reader sensitivity. AI_{spec}=artificial intelligence score cut-off point matched at mean first reader specificity.

Supplementary eTable 5: Detection accuracy analysis with inclusion of next-round screen-detected cancers and long-term cancers in both study scenarios

	Sensitivity (95% CI); p value*	Specificity (95% CI); p value*	PPV (95% CI); p value†	NPV (95% CI); p value†
**Standalone AI**				
First reader	30.5 (29.2-31.8); ref.	97.8 (97.7-97.9); ref.	20.2 (19.3-21.2); ref.	98.7 (98.7-98.8); ref.
Standalone AI_{sens}	37.7 (36.2-39.2); <0.0001	96.6 (96.5-96.7); <0.0001	16.9 (16.1-17.7); <0.0001	98.8 (98.8-98.9); <0.0001
Standalone AI_{spec}	33.3 (31.8-34.7); <0.0001	97.9 (97.8-98.0); 0.03	22.4 (21.3-23.4); <0.0001	98.8 (98.7-98.8); 0.02
**AI-integrated screening**				
Combined reading	34.5 (33.2-35.8); ref.	97.9 (97.9-98.0); ref.	23.2 (22.2-24.3); ref.	98.8 (98.7-98.8); ref.
Integrated AI_{sens}	39.1 (37.7-40.6); <0.0001	97.3 (97.3-97.4); <0.0001	21.1 (20.2-22.0); <0.0001	98.9 (98.8-98.9); 0.0004
Integrated AI_{spec}	37.3 (35.9-38.7); <0.0001	97.9 (97.9-98.0); 0.28	24.9 (23.9-26.0); 0.001	98.8 (98.8-98.9); 0.02

Data are % (95% CI); p value. Next-round screen-detected cancers (n=1232) and long-term cancers (n=2372) include ductal carcinoma in situ. Linear regression with the measure of performance was used to take the correlation between women and possible multiple cancers into account. PPV=positive predictive value. NPV=negative predictive value. AI_{sens}=artificial intelligence score cut-off point matched at mean first reader sensitivity from the primary analysis presented in study table 2. AI_{spec}=artificial intelligence score cut-off point matched at mean first reader specificity from the primary analysis presented in study table 2. *p values were calculated using McNemar's test. †p values were calculated using exact binomial test.
